# Automatic Cone Photoreceptor Localisation in Healthy and Stargardt Afflicted Retinas Using Deep Learning

**DOI:** 10.1038/s41598-018-26350-3

**Published:** 2018-05-21

**Authors:** Benjamin Davidson, Angelos Kalitzeos, Joseph Carroll, Alfredo Dubra, Sebastien Ourselin, Michel Michaelides, Christos Bergeles

**Affiliations:** 1 Welcome/EPSRC Centre for Interventional and Surgical Sciences, London, UCL UK; 2 Translational Imaging Group, Centre for Medical Image Computing, London, UCL UK; 3 NIHR Biomedical Research Centre, Moorfields Eye Hospital and Institute of Ophthalmology, London, UCL UK; 4 Medical College of Wisconsin, Milwaukee, WI USA; 5 Stanford University, Stanford, CA USA

## Abstract

We present a robust deep learning framework for the automatic localisation of cone photoreceptor cells in Adaptive Optics Scanning Light Ophthalmoscope (AOSLO) split-detection images. Monitoring cone photoreceptors with AOSLO imaging grants an excellent view into retinal structure and health, provides new perspectives into well known pathologies, and allows clinicians to monitor the effectiveness of experimental treatments. The MultiDimensional Recurrent Neural Network (MDRNN) approach developed in this paper is the first method capable of reliably and automatically identifying cones in both healthy retinas and retinas afflicted with Stargardt disease. Therefore, it represents a leap forward in the computational image processing of AOSLO images, and can provide clinical support in on-going longitudinal studies of disease progression and therapy. We validate our method using images from healthy subjects and subjects with the inherited retinal pathology Stargardt disease, which significantly alters image quality and cone density. We conduct a thorough comparison of our method with current state-of-the-art methods, and demonstrate that the proposed approach is both more accurate and appreciably faster in localizing cones. As further validation to the method’s robustness, we demonstrate it can be successfully applied to images of retinas with pathologies not present in the training data: achromatopsia, and retinitis pigmentosa.

## Introduction

Adaptive Optics Scanning Light Ophthalmoscopy (AOSLO) is an optical imaging technique that eliminates aberration-induced distortion in retinal images, allowing for high resolution, *in vivo* imaging of the photoreceptor layer of the retina^1^. To achieve this, an adaptive optics (AO) system is embedded within a scanning light ophthalmoscope (SLO)^2^. AO is a technique by which, through a wavefront sensor and actuated mirror, wavefront aberrations, present due to the inhomogeneous medium of the eye, are measured and then dynamically compensated for. As such, AO can be applied to any ophthalmic imaging device which requires passing light into or out of the eye, but is typically used with SLOs as these produce the best contrast and highest resolution^1^. Furthermore, modern AOSLO imaging captures 3 channels simultaneously (confocal, split-detection, and dark-field), with each highlighting different retinal structures. In this work we focus on the automated image analysis of the split-detection channel, which has been shown to improve photoreceptor identification in retinas afflicted with pathology^2,3^.

AOSLO split-detection images are currently manually analysed to extract the location of cone photoreceptor cells within the images. Cone photoreceptors (cone photoreceptors will be referred to as cones through the manuscript) are the cells responsible for our acute, day-time vision. The ability to quantitatively assess the cone mosaic through AOSLO imaging provides new insights into well-studied pathologies and into the therapeutic effect of experimental treatments^1,3^. The laborious nature of manually locating the thousands of cones within all acquired AOSLO images, however, is a severe bottleneck in the application of this technology to larger studies^3^. By automating the cone localisation process, we are pushing AOSLO towards mainstream clinical use.

**Figure 1:**
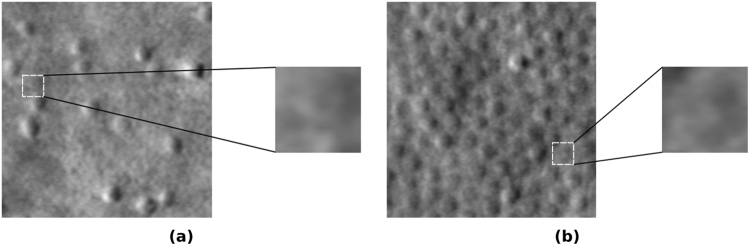
The two distinct image types: (**a**) an image of a retina afflicted by Stargardt disease, and (**b**) an image of a healthy retina. When image patches are considered in isolation, they cannot always be reliably classified. However, when global context is considered (larger images) it is obvious that the patch in (**a**) is not a cone, but the patch in (**b**) is a cone.

There has already been extensive research on automating cone localisation in images of healthy retinas, with state-of-the-art algorithms obtaining similar-to-human performance^4–7^. Only recently, however, have researchers attempted to tackle the problem of automatically detecting photoreceptors in images acquired from retinas afflicted with pathologies. There, it proves that the problem is significantly more challenging, primarily due to a lack of a regularly appearing cone mosaic, and the acquisition of images of a reduced quality, due to the inability of disease-afflicted subjects to fixate well (see Fig. 1). Only a handful of manuscripts have presented promising results on diseased images^4^. Even there, however, the algorithms significantly under-perform when compared to human graders. This discrepancy in performance when considering images of healthy retinas versus ones with pathology must be addressed for automatic localisation tools to be of clinical use.

Towards overcoming this discrepancy, we adopt a deep learning framework to locate cone centroids in AOSLO split-detection images. Specifically, a combination of a MultiDimensional Recurrent Neural Network^8^ and convolutional layers^9^ is used to semantically segment AOSLO split-detection images into two classes: cone and background.

The innovative introduction of MDRNNs allows the network to consider the entire image whilst classifying a single pixel, as well as being able to take advantage of the highly correlated classifications of neighbouring pixels. This is in contrast to the deep learning approach presented in Cunefare *et al*.^7^, which uses a sliding-window convolutional network. This approach classifies pixels based only on the examined sliding-window patch, *i.e*. considering only local information, and cannot make use of global image features. The use of global context is, however, critical for accurate segmentations of healthy retinas and those with pathology, as it is often difficult to classify pixels when only considering local information (see Fig. 1). By taking advantage of global image features and the correlated classifications of neighbouring pixels, our network achieved a highly accurate model of cone appearance, leading to reliable segmentations.

MDRNNs offer benefits over sliding window classifiers, and have also shown themselves to be superior to fully convolutional segmentation frameworks in many instances^10–13^. One of the main difficulties in semantic segmentation is simultaneously capturing global and local information. In fully convolutional networks the global information is gathered by creating a large receptive field, with most of the state-of-the-art convolutional segmentation frameworks^14,15^ relying on very deep networks, such as ResNet^16^ or VGG net^17^, to achieve this. However, the empirical receptive field of such networks has been shown to be smaller than that required to capture global context^18^. In contrast to this, the components making up MDRNNs have been shown to reliably enable global information to be available at each pixel^19^. Furthermore, there is no mechanism for enforcing the consistency of neighbouring pixel classifications in most of the fully convolutional approaches. In contrast to this, MDRNNs have access to neighbouring features when classifying a pixel and can therefore learn the required, consistent classifications. There are a number of examples of recurrent architectures outperforming fully convolutional networks in scene segmentation tasks. In these architectures, recurrent networks are used to either give the network access to global context, or ensure consistency between pixel classifications. Both of these architectural strengths are critical for accurate AOSLO split-detection segmentation, and so we opted to use MDRNNs as our segmentation framework.

In what follows, we present more details on the proposed deep learning algorithm. A detailed comparison of our approach with the state-of-the-art^4,7^ methods on images from healthy volunteers and of volunteers afflicted by Stargardt disease demonstrates that the proposed MDRNN framework is more accurate and significantly faster. Following this we show, qualitatively, that, in some cases, the network is able to generalise to images of retinas with pathologies that were not present in the training set, retinitis pigmentosa and achromatopsia.

The research study presented here was conducted in accordance with the tenets of the Declaration of Helsinki (1983 Revision) and the applicable regulatory requirements. The study and its procedures were approved by the ethics committees of Moorfields Eye Hospital and University College London. All participants provided their informed consent in order to enrol.

## Methods

**Figure 2:**
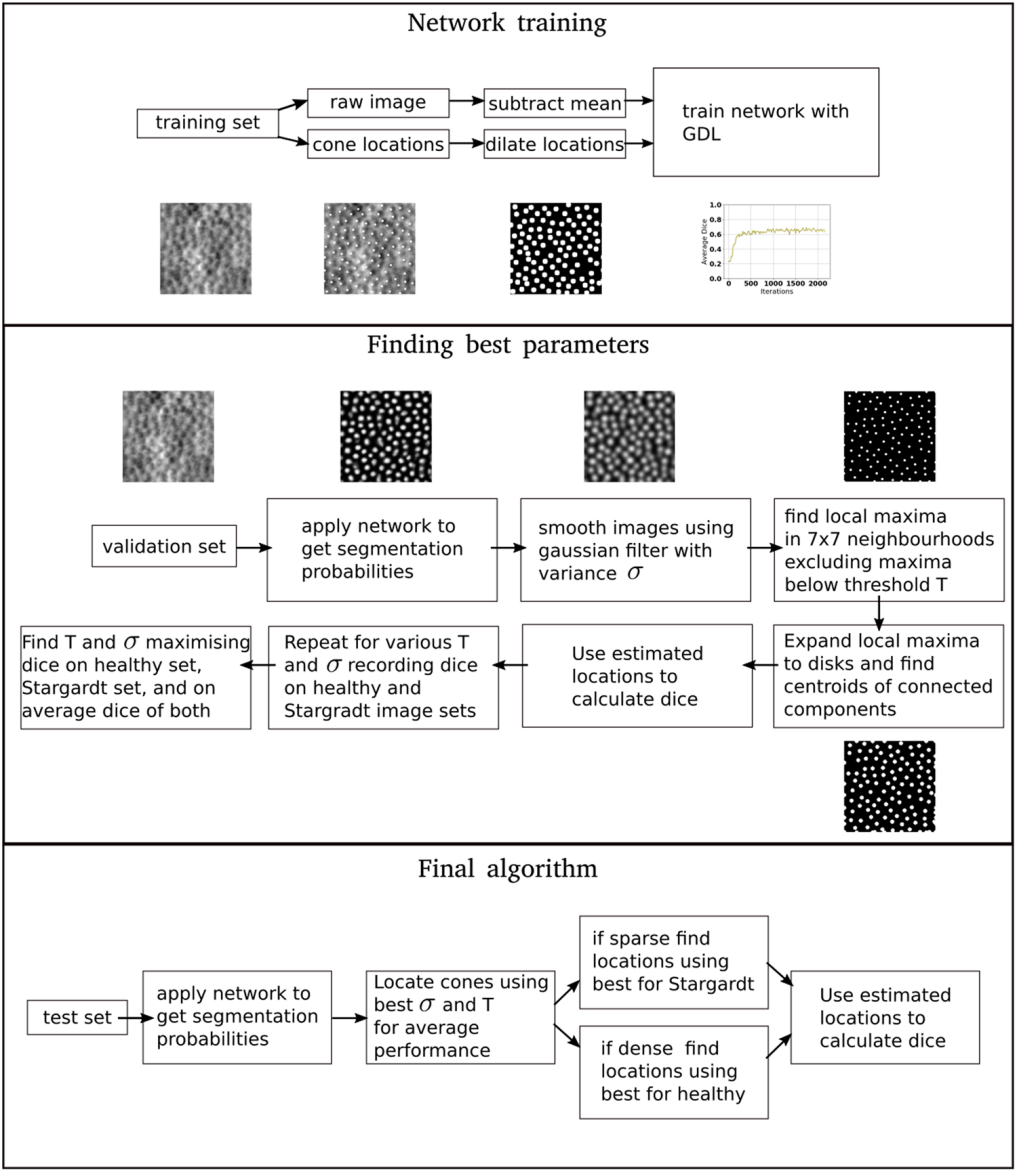
Detailed breakdown of entire approach.

An overview of the method is as follows. MDRNN and convolutional layers were stacked into a single segmentation network. This was trained using manually generated segmentations. To overcome class imbalances, the Generalised Dice Loss (GDL) was used as the objective function^20^. The trained network could then assign a probability to each pixel in an image, representing the probability that the pixel belonged to a cone. Local maxima were found amongst these probabilities, and taken to be the centres of cones within the image (see Fig. 2).

### Data Pre-Processing

The segmentation network learned how to classify pixels through supervised learning. The network was presented with images *I* and their corresponding segmentation *S*_*I*_,

1 $$(I-{\rm{mean}}(I),{S}_{I})$$

where *S*_*I*_ is a 2D binary mask of the same dimensions as *I*, with a one-hot vector indicating a pixel’s class at each point of the grid. We centred the image *I* by subtracting its scalar mean, as is standard in segmentation networks^12,14^. However, we did not normalise the variance as early experiments showed that this has no effect on performance. The segmentations *S*_*I*_ were created by an expert grader manually locating cones within images, and then dilating the locations to disks of a manually chosen, fixed radius *r*_*I*_. The radius *r*_*I*_ was chosen so that when dilating each location to a disk of size *r*_*I*_, the borders of cones in the image were still visible with the disk covering as much of the cone as possible. Note that disk size was constant for a single image, but could vary between images. The data available were 290 images (and their segmentations), corresponding to 142 healthy retinas and 148 retinas afflicted by Stargardt disease, acquired from 8 subjects with Stargardt disease, and 17 subjects with no pathology. These data consisted of the same datasets used in Bergeles *et al*.^4^ and Cunefare *et al*.^6^ with some additional images acquired using an AOSLO setup described in Scoles *et al*.^2^. The images in Bergeles *et al*.^4^ were chosen by hand to cover a range of eccentricities, from various subjects. Images from the Cunefare *et al*.^6^ dataset, were randomly sampled from multiple eccentricities. Their size was chosen so that they would contain roughly 100 cones. The additional data were chosen only under the condition that cones be resolvable within each image. In every image, all cones in the image were manually located. The same experienced member of the clinical team was used to mark all images acquired at Moorfields Eye Hospital, whilst a single expert grader was used for the Cunefare *et al*.^6^ dataset. All images had a field-of-view of either 1 × 1deg or 1.5 × 1.5 deg; covered a range of eccentricities along all meridians (300–2800 *μm*); and contained up to 470504 ≈ 686 × 686 pixels and at least 30625 = 175 × 175 pixels.

The data was split into 176 images for training (87 healthy, 93 Stargardt-afflicted), 14 for validation (7 healthy, 7 Stargardt-afflicted) and 96 for testing (48 healthy, 48 Stargardt-afflicted). There was no overlap in subject data between the testing set and training plus validation set; there was overlap between training and validation. The breakdown of subjects was as follows: for training 11 healthy and 2 with Stargardt; for validation 2 healthy and 2 with Stargardt; and for testing 6 healthy and 5 with Stargardt. This partition was chosen to maximise the amount of data available for training and testing, whilst not positively biasing the performance on the test set.

### Code Availability

The software will be made available for research purposes upon request to the corresponding author.

### Data Availability

The datasets generated during and/or analysed during the current study are not publicly available due to restrictions with regards to exchanging patient information and data in the United Kingdom. The authors, however, will accommodate reasonable requests through material transfer agreements.

## Network Components

### Multidimensional Recurrent Neural Networks

MDRNN^8^ layers were used throughout the segmentation network to capture global context, and make use of the highly correlated classifications. MDRNNs are a natural extension to recurrent neural networks (RNNs), which incorporate the multidimensional structure of data that is typically lost when applying RNNs to multidimensional data. By doing so, MDRNNs can more easily learn long and short range dependencies between pixels, and can appreciate both the global context of a pixel and its immediate, local context.

To illustrate the benefit of applying MDRNNs to image data in comparison to RNNs, we briefly describe how RNNs are typically applied to images. Under the standard RNN framework, a *k*-channel image *I* is converted to a 1. D sequence $({I}_{0},\,{I}_{1},\,\ldots ,\,{I}_{hw-1})$ of pixels, and a *recurrent block* applies (2) to the sequence, iteratively generating activations *h*_*i*_ from the preceding activation *h*_*i−*1_ and current pixel *I*_*i*_:

2 $${{\rm{block}}}_{A,B}({I}_{i},\,{h}_{i-1})=tanh\,(A{I}_{i}+B{h}_{i-1})={h}_{i},\,{\rm{where}}\,{h}_{-1}=\mathrm{0,}\,\,\,\,A\in {{\mathbb{R}}}^{u\times k},\,\,\,\,B\in {{\mathbb{R}}}^{u\times u}\mathrm{.}$$

**Figure 3:**
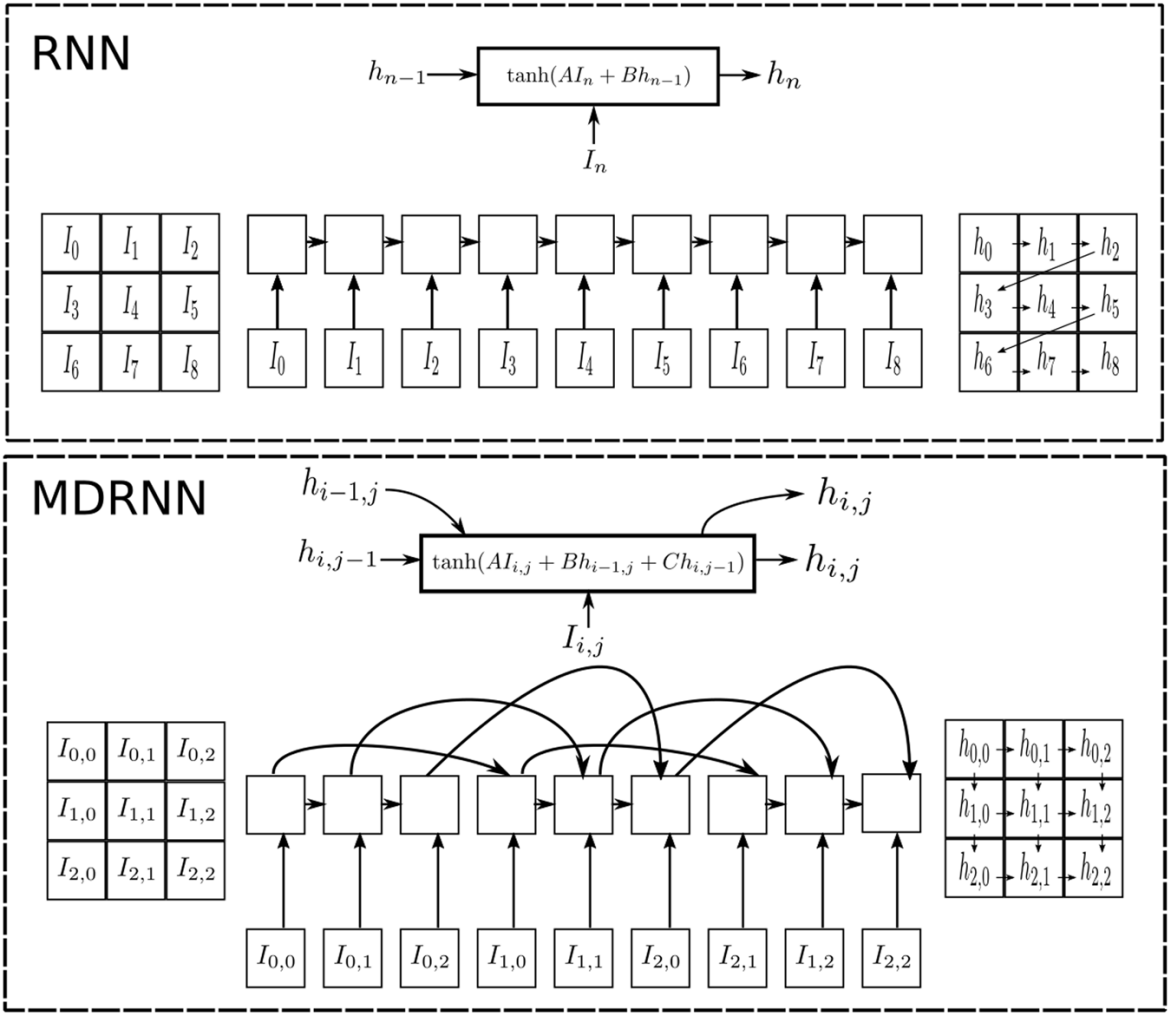
These diagrams showcase the construction of activations from previous activations and pixels for the case of RNN and MDRNN. Note that the zero vector is used when previous activations are not available, *e.g*. ${h}_{-1}=\mathrm{0,}\,{h}_{-\mathrm{1,}j}=0$ and ${h}_{i,-1}=0$. The grids on the right demonstrate the linking of those activations. In the RNN, an arrow from *h*_*i*_ to *h*_*j*_ indicates that *h*_*i*_ was used in the construction of *h*_*j*_, *i.e*. ${h}_{j}=tanh(A{I}_{i}+B{h}_{i})$. Note that the distance that activations need to travel before being used as context for nearby pixels differs significantly between the two approaches. For example, *h*_2_ must pass through 6 recurrent blocks before being used as context for *h*_8_ in the RNN, whilst ${h}_{\mathrm{0,2}}$ only requires 2 steps before being available as context in the MDRNN.

Note that *u* is a hyperparameter known as the number of units. The block in (2) can learn dependencies between pixels through the recurrent connection $B{h}_{i-1}$, which enforces a dependency between activations and introduces previous activations as context. The limitation of the application of RNNs on multidimensional (e.g. 2D) data, is that activations must travel through many recurrent blocks before being available as context (see Fig. 3). This makes dependency-learning challenging and negatively affects network performance.

To rectify this, MDRNNs maintain the spatial relationship of pixel data in *I* by modifying the recurrent block to accept two recurrent connections, as in

3 $${{\rm{block}}}_{A,B,C}({I}_{i,j},\,{h}_{i-\mathrm{1,}j},\,{h}_{i,j-1})=tanh(A{I}_{i,j}+B{h}_{i-\mathrm{1,}j}+C{h}_{i,j-1})={h}_{i,j},$$

where

4 $${h}_{r,s}=0\,{\rm{if}}\,r < 0\,{\rm{or}}\,s < 0\,A\in {{\mathbb{R}}}^{u\times k},\,B\in {{\mathbb{R}}}^{u\times u},\,\,C\in {{\mathbb{R}}}^{u\times u}\mathrm{.}$$

Equation (3) iteratively produces activations *h*_*i*,*j*_ by using both *h*_*i*−1,*j*_ and *h*_*i*,*j*−*1*_ together with the current pixel *I*_*i*,*j*_. The recurrent connections in the multidimensional case allow activations to be used as context without having to traverse numerous recurrent blocks (see Fig. 3). This facilitates dependency learning and the use of global context.

Multi-Dimensional Long-Short Term Memory (MDLSTM) blocks^8^ were used in our framework to avoid the common vanishing gradients problem often encountered when recurrent networks are applied to large sequences. With MDLSTM blocks, long-range dependencies, spanning possibly thousands of steps, critical to utilising global context, can be learned. This allows context from the entire image to be used during the local decision at a single pixel^19,21^.

**Figure 4:**
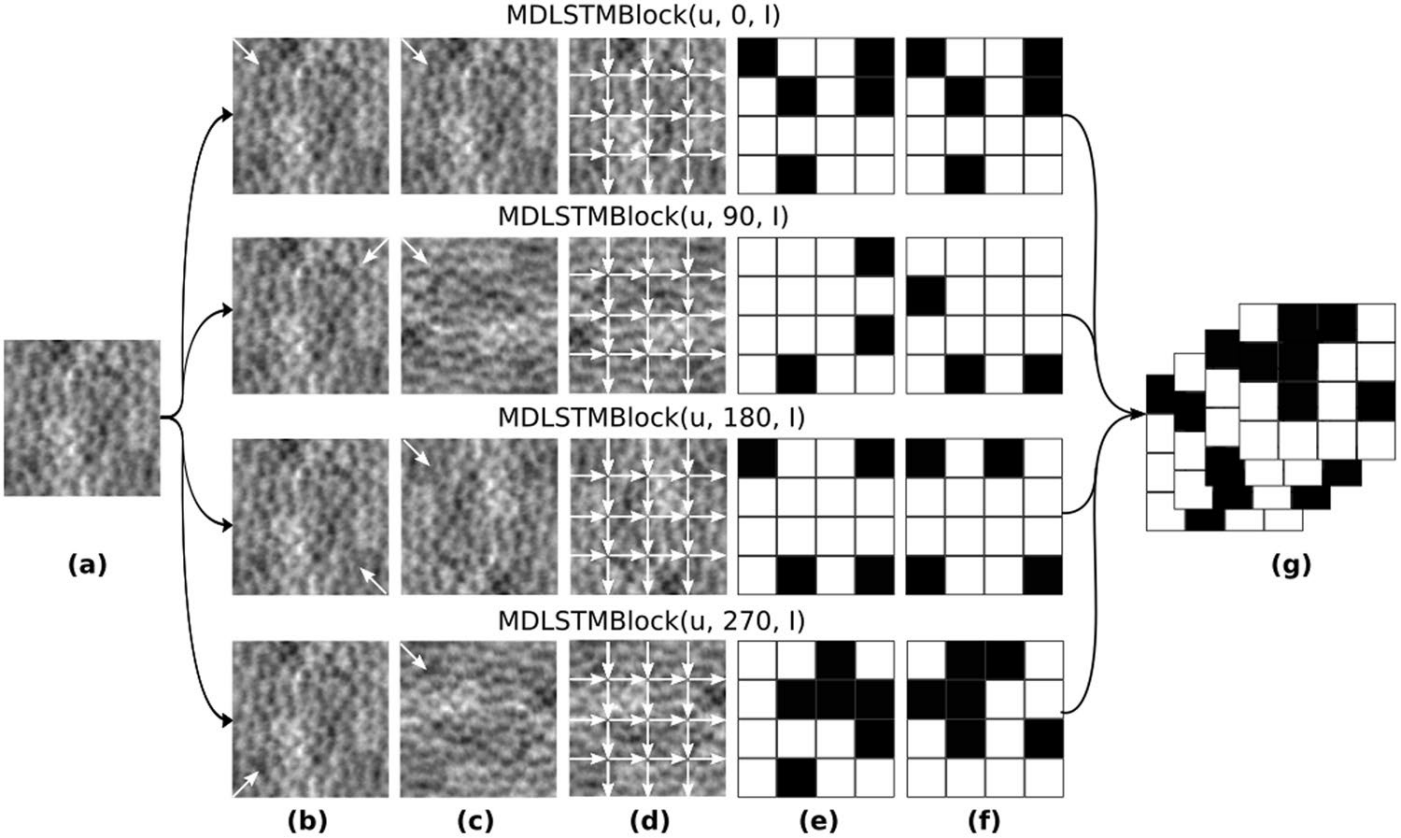
Each row shows an application of a different MDLSTM block. Each block processes the input pixels in a single direction, indicated by the arrow originating from the corner. To process pixels in four different orders, we rotate image *I* by a chosen multiple of 90 degrees. Pixels in the rotated image are then processed in a fixed order: from top to bottom, left to right. After rotating and processing the image the activations associated to each pixel are rotated back so that they are aligned in a feature image *h*, where features ${h}_{i,j}\in {{\mathbb{R}}}^{u}$ correspond to pixels ${I}_{i,j}$. (**a**) Image *I*; (**b**) direction pixels will be processed in by the block. For example, in the third row, pixels from the unrotated image, will be processed bottom to top, right to left; (**c**) direction to process pixels in the rotated image to achieve an equivalent processing order as in (**b**,**d**) fixed processing order, top to bottom, left to right; (**e**) produced activations; (f) activations after applying the inverse rotation; (**g**) feature image *h*.

The specific implementation of MDLSTM blocks used in this paper is provided in Algorithms 1 and 2. Algorithm 1 constructs activations by rotating an input *k*-channel image and applying a MDLSTM block, pixel by pixel, from top to bottom, left to right. Of note here is the cell state computation in equation (10)^22^. This prevents the cell state from growing unbounded, a problem which MDLSTMs often face, and which makes training difficult. Rotating the image before applying the block has the same effect as processing the pixels in a different order and ensures that the network has access to a truly global context (see Fig. 4). The four rotations are used as these are the minimum number of rotations required so that each stacked feature vector has accumulated context from the entire image, see Graves *et al*.^8^ for details. Algorithm 2 shows how 4 distinct MDLSTM blocks are used within a single MDLSTM layer to process the image using 4 different directions. Finally, to speed up training, we used a parallel implementation of Algorithms 1 and 2^23^.

**Algorithm 1:**
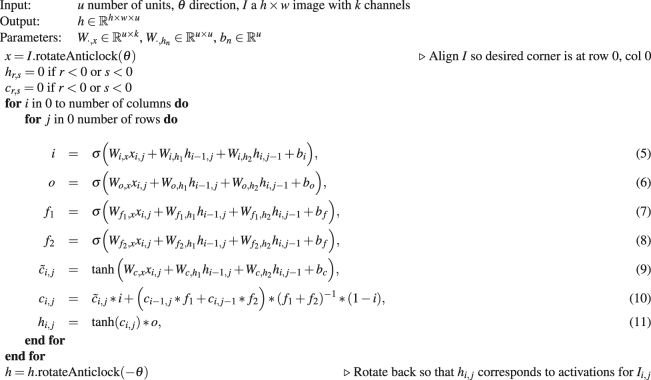
MDLSTMBlock, where * denotes element-wise multiplication and $\sigma (x\mathrm{)=(1}+\exp (-\,x{))}^{-1}$.

**Algorithm 2:**
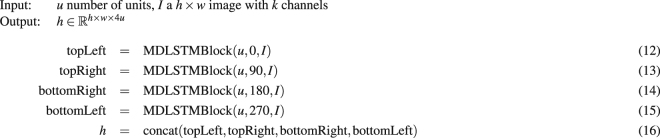
MDLSTMLayer.

### Convolutional and Fully Connected Layers

Convolutional layers were used to take a weighted average of the intermediate output features from preceding MDLSTM layers. These output features tend to aggregate higher level image features, which when combined can form even higher level image features. This process continues until we have the features cone, or not cone. Aggregating the intermediate features meant each application of an MDLSTM block to a single pixel required 10592 multiplications, as opposed to the 30912 it would take, were we to keep each output feature as an input channel. The result of this was faster network training, which allowed us to stack many MDLSTM layers on top of one another. Convolutional layers excel at utilising local context. Intermediate features typically highlight meaningful structures. Therefore, with MDLSTMs capability of utilising global context, and convolutional layers strength in detecting pertinent local features, we were able to build a highly accurate model of cone appearance. In every convolutional layer, each filter was 3 × 3 pixels, with a tanh non-linearity, and all inputs were padded with zeros so that the height and width dimensions of the output are the same as the input (see Table 1).

**Table 1:** Segmentation network, where b is the batch size and h, w is the size of image patch extracted from AOSLO images.

Layer	Input size	Output size
3 × 3 convolution	(*b*, *h*, *w*, 1)	(*b*, *h*, *w*, 1)
32 unit MDLSTM	(*b*, *h*, *w*, 1)	(*b*, *h*, *w*, 4*32)
3 × 3 convolution	(*b*, *h*, *w*, 4*32)	(*b*, *h*, *w*, 1)
32 unit MDLSTM	(*b*, *h*, *w*, 1)	(*b*, *h*, *w*, 4*32)
Fully connected - hidden	(*b*, *h*, *w*, 4*32)	(*b*, *h*, *w*, 64)
Fully connected - output	(*b*, *h*, *w*, 64)	(*b*, *h*, *w*, 2)
Softmax	(*b*, *h*, *w*, 2)	(*b*, *h*, *w*, 2)

The final layers of our network were a fully connected layer, and a softmax layer. The input to the fully connected layer was always a *h* × *w*, *n*-channel image, where (*h*, *w*) were the dimensions of the input AOSLO image, and *n* the number of output activations for each pixel. Each pixel’s *n* activations were processed by the same fully connected layer with 64 hidden units, ReLU activations, and 2 output neurons, one for each class (cone and background). A softmax layer was then used to transform these 2 outputs into probabilities.

### Complete Network

**Figure 5:**
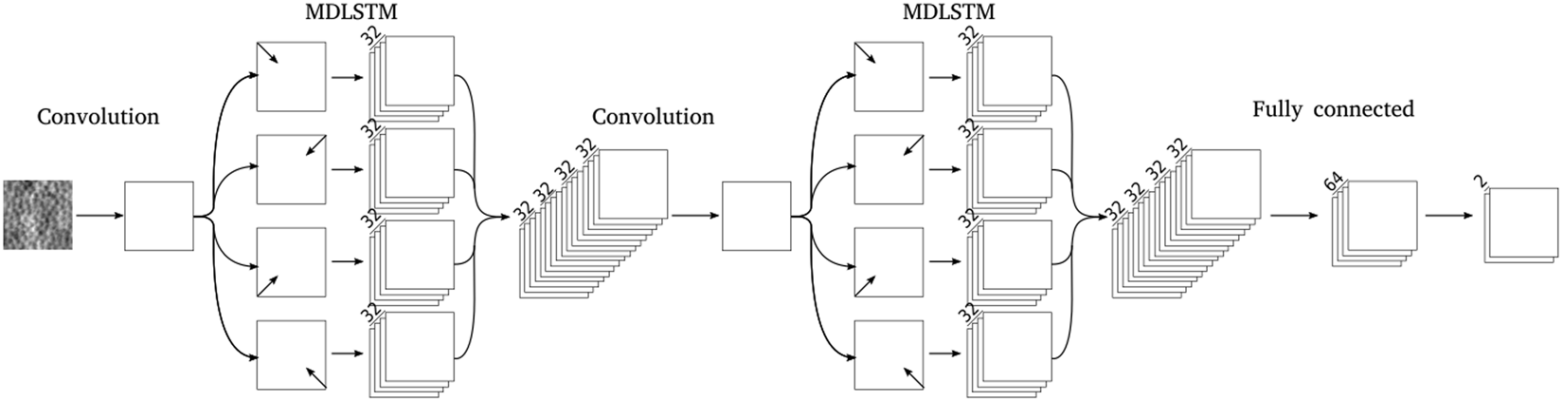
A visualisation of the proposed Neural Network architecture, showing, in order, the following layers: MDLSTM, convolution, MDLSTM, and fully connected. In each MDLSTM layer, the four MDLSTM blocks, which process the input in four different directions, are depicted as four arrows highlighting the direction each processes pixels in.

The full architecture of our network is as follows. Following the input layer, in order there is: a convolutional layer, MDLSTM layer, convolutional layer, MDLSTM layer, and finally, a fully connected layer. The fully connected layer’s outputs are transformed to probabilities using the softmax function (see Table 1 and Fig. 5).

### Training

Gradient-based methods were used to train the network. First, weights were randomly initialised: MDLSTM weights were sampled from a normal distribution with mean *μ* = 0 and variance *σ* = 0.25, convolutional filters were uniformly sampled over [−0.1, 0.1], and all biases were initialised to zero. The Gaussian weights were chosen through preliminary experimentation to boost training. Namely, if large initial values were used, the gradients would explode; for smaller gradient values the network would make activations vanishingly small. To update the weights, we used backpropogation^24^ and the RMS optimiser^25^ to minimise the loss. Since the RMS optimiser has been successfully applied to MDLSTM architectures for medical image segmentation in the literature^12^, it was also employed here. The default hyperparameters of Tensorflow^26^ version 1.2.0 were used (Learning rate of 0.001, decay of 0.9, and momentum of 0).

In AOSLO split-detection images the background is the overwhelming majority class. This poses a problem when using gradient-based learning methods, which will find local optima and classify everything as background. To overcome this class imbalance, the network was trained using the Generalised Dice Loss (GDL), which has been shown to handle imbalanced classes in segmentation tasks^20^.

The GDL was calculated as follows. Let ${\hat{b}}_{i}$, and ${\hat{c}}_{i}$, be the estimated probability of pixel *i* being background or cone respectively (so ${\hat{b}}_{i}=1-{\hat{c}}_{i}$). Furthermore, let *b*_*i*_ and c_*i*_ be the true probability. Then, the GDL for a single image *I*, is given by:

17 $$1-2\frac{\sum _{x\in \{b,c\}}{w}_{x}\sum _{i\in I}{x}_{i}{\hat{x}}_{i}}{\sum _{x\in \{b,c\}}{w}_{x}\sum _{i\in I}{x}_{i}+{\hat{x}}_{i}},\,\mathrm{where},\,{w}_{x}=\mathrm{1/}{(\sum _{i\in I}{x}_{i})}^{2}\mathrm{.}$$

When considering a batch, the loss was taken as the average GDL over all images in the batch.

The network was always trained in minibatches of size 8, where each example was a random 128 × 128 crop from an image. This allowed us to stay within the memory limitations of a 4GB GPU while training. Note that despite training on 128 × 128 patches, the trained network can be applied to arbitrary sized images *h* × *w* (*h* and *w* are independent from the parameter weights in Algorithm 1).

Early stopping was used as a form of regularisation, where training ceased after there had been no improvement on the validation set for 20 epochs^27^.

### Cone Centroid Recovery

To recover cone centroids from the segmentation probabilities, locally maximal probabilities were found and selected as the desired cone centroids. After training, the network was able to process an image $I\in {{\mathbb{R}}}^{h\times w}$ to generate a probability map ${\rm{Net}}(I)\in {\mathrm{[0,}\mathrm{1]}}^{h\times w}$, where

18 $${\rm{Net}}{(I)}_{i,j}={\rm{probability}}({{\rm{pixel}}}_{i,j}\,{\rm{is}}\,{\rm{a}}\,{\rm{cone}}\mathrm{).}$$

To recover the centroids we:

- Smoothed Net(*I*) with a Gaussian filter of variance *σ*, to diminish isolated, large probabilities;
- Found local maxima in the smoothed Net(*I*), over neighborhoods of size 7 × 7, as the larger neighbourhood helped to eliminate local maxima resulting by noise;
- Rejected local maxima below a threshold *T*, so that the identified local maxima were actually cones, and not just the most cone-looking background pixels;
- Rejected local maxima within 7 pixels of the border, to avoid border artefacts;
- Combined neigbouring centroids that are less than 8-pixels apart into a single centroid^28^;

The validation set was used to identify σ and *T* that maximised the performance on images of healthy retinas, retinas afflicted with disease, and on both diseased and healthy images when considered simultaneously. These “ideal” parameters are indicated as $({\sigma }_{h},{T}_{h}),({\sigma }_{s},{T}_{s})$ and $({\sigma }_{b},{T}_{b})$ respectively, in the following.

The final algorithm for localisation used these three sets of parameters to locate cones. First, $({\sigma }_{b},{T}_{b})$ were used to determine if an image was densely or sparsely populated with cones. An image was considered densely populated if more than 0.0011 cones per pixel were found. This cut-off was chosen as it was the upper bound of a 95% confidence interval for the number of cones-per-pixel in images of retinas with Stargardt disease on the validation set. Following the dense-versus-sparse classification, the image was reprocessed using the appropriate set of parameters, i.e. $({\sigma }_{h},{T}_{h})$ if it was densely populated, and $({\sigma }_{s},{T}_{s})$ otherwise (see Fig. 2).

### Evaluation Metric

Our method is validated against the gold-standard of a trained grader manually locating cones. This is taken as the gold-standard as previous work has shown that manually locating cones in healthy eyes, and eyes with Stargardt, is reliable and repeatable^29–32^.

The performance of the network is evaluated using the Dice coefficient^33^. This is a single-number metric encompassing: true positives (TP), *i.e*. cones which both the expert grader and the network located; false positives (FP), *i.e*. cones which only the network located; and false negatives (FN), *i.e*. cones the expert grader located, but the network failed to locate. We considered an estimated cone centroid as a true positive if it was within ${\rm{m}}{\rm{i}}{\rm{n}}\mathrm{(0.75}d,\,\mathrm{20)}$ pixels of an actual centroid, where *d* was the median cone spacing in the given image. This is similar to the approach in Cunefare *et al*.^7^, where we added the maximum distance of 20 pixels since the median distance between cones in retinas with Stargardt disease may be very large. Every estimated cone could only be matched to a single, actual cone location, and we always considered as the match the estimated location which was closest to the detection. The Dice calculation itself is straight-forward and is given by

19 $${\rm{Dice}}=\frac{2{\rm{TP}}}{2{\rm{TP}}+{\rm{FP}}+{\rm{FN}}}\mathrm{.}$$

### Comparison to State-of-the-Art

We compare our method with the approaches of Bergeles *et al*.^4^ and Cunefare *et al*.^7^. For a fair comparison of our method to that presented in Cunefare *et al*.^7^, we retrained the convolutional network on the dataset used herein. Further, we retain the same test sets and evaluation for our comparison to the convolutional approach.

## Results

### Experiments

**Figure 6:**
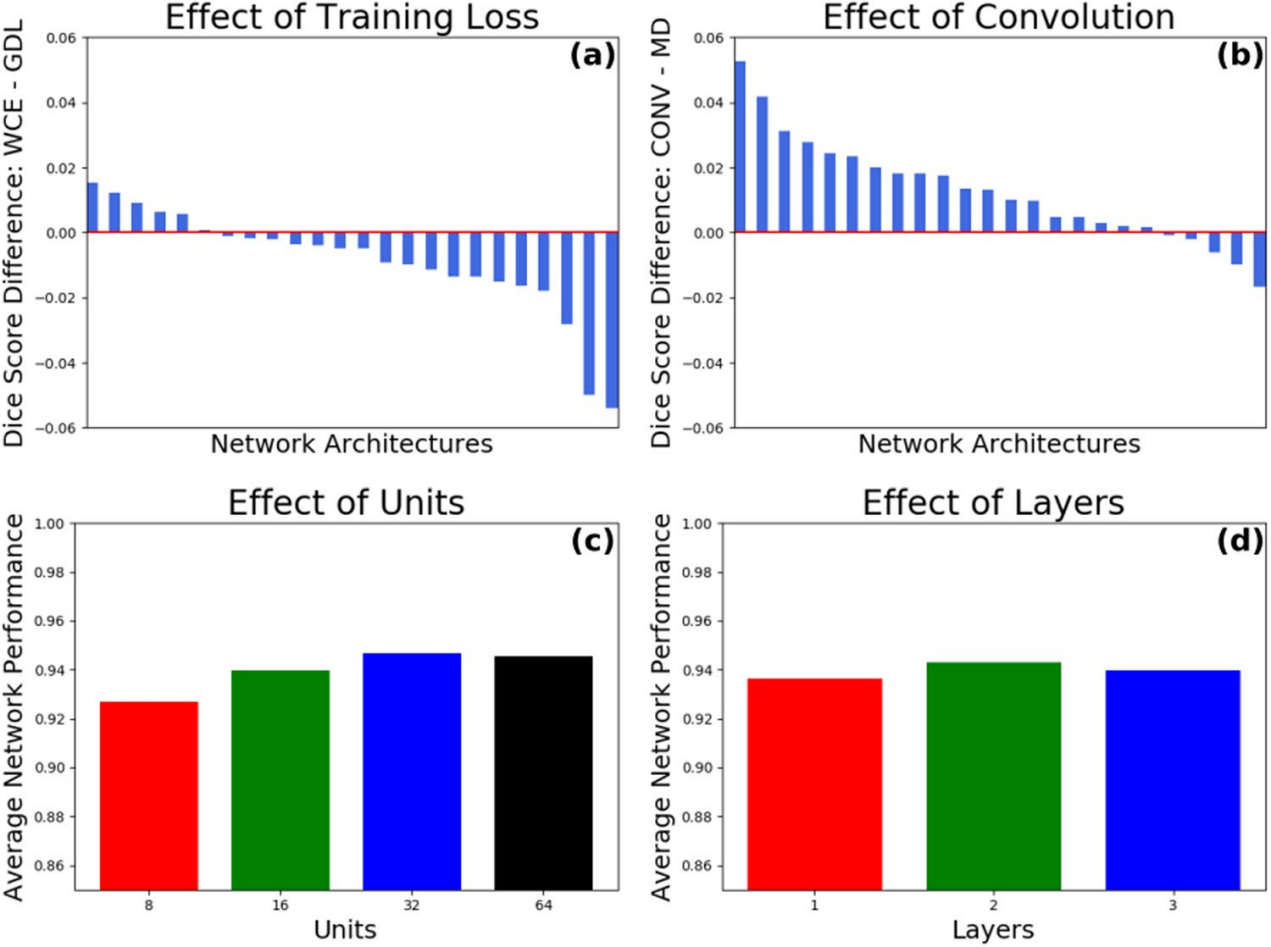
The above figure shows the basis upon which the final network architecture was chosen. (**a**) Shows that using the GDL is superior to the WCE; (**b**) shows that having a convolutional layer as the first layer is superior to an MDLSTM layer; (**c**) and (**d**) show that using 32 units and 2 layers, respectively, produces, on average, the strongest network.

As a first step, many configurations of MDLSTM and convolutional layers were evaluated on the validation set in order to understand the effect that the number of layers and units has on network performance. In initial experiments we utilised a training regime as in Havaei *et al*.^14^, which used a 2-phase training and Weighted Cross Entropy (WCE). However, we found that this performed poorly in comparison to the GDL, as previous work has shown^20^ (see Fig. 6). Following training, network performance was evaluated by calculating the average Dice score on images from the validation set (see Table 2).

**Table 2:** Each network comprises convolutional layers denoted by *C*, MDLSTM layers denoted by *M*, and a fully connected layer *F*.

Architecture	8 units	16 units	32 units	64 units
C-M-F	0.9410	0.9504	0.9439	0.9468
C-M-C-M-F	0.9456	0.9532	0.9553	0.9504
C-M-C-M-C-M-F	0.9465	0.9489	0.9507	0.9499
M-F	0.9176	0.9225	0.9390	0.9565
M-C-M-F	0.9282	0.9352	0.9418	0.9408
M-C-M-C-M-F	0.9474	0.9388	0.9569	0.9518

For example, *C*-*M*-*F* has a convolutional layer as its first layer, followed by an MDLSTM layer, and finally the fully connected layer. Every network architecture was followed by a softmax, each of the MDLSTM layers had the same number of units, and each convolutional layer was as described previously.

From the experiments we concluded that a 32 unit, 2 layer network, and a convolutional layer following the input layer to be the most suited for our segmentation task. By averaging the performance of all *n*-unit networks, we found that the 32-unit network performed best. Similarly, the average performance of 2 layer networks was found to be the highest. And finally, when comparing networks with a convolutional layer after the input layer, to those without, we found that a preceding convolutional layer led to improved performance (see Fig. 6). Based on these observations the network architecture was chosen as previously described. Ten identical networks, of 32 units, 2 layers and a preceding convolutional layer, were trained and the highest performing network was then evaluated on the test set. The best performing network achieved an average Dice score of 0.9577 on the validation set.

### Performance of Algorithm

In Table 3 we present the performance of our method in comparison to the state-of-the-art approaches^4,7^. Experiments show that the proposed method is the most robust, with more accurate localisations over both image types, despite optimising the sliding-window approach^7^ to the same dataset. In addition, Table 3 indicates that our approach has similar performance to the sliding-window network when healthy images are considered. This supports the algorithm’s robustness, as it is able to compete with a method tailored specifically for images of healthy cones, whilst itself being optimised for joint performance over a wide array of inputs. Table 3 also shows that when considering average performance on both image sets, the proposed approach has a reduced variance, further highlighting its robustness. Finally, we see that there is overfitting to the validation set as the best performing network achieves an accuracy of 0.9577 on the validation set, but 0.9431 on the test set. This is to be expected when only a reduced set of 14 images is used for validation. Figure 8 shows examples of our method’s performance on the test set.

**Table 3:** Dice scores and respective standard deviations for the proposed MDRNN method and the current state-of-the-art automatic detection methods.

Test Performance	Healthy	Stargardt	Average
Proposed	0.9628 ± 0.0252	**09233 ± 0.0571**	**0.9431 ± 0.0482**
Cunefare *et al*.^7^ retrained	0.9540 ± 0.0328	0.8797 ± 0.1051	0.9168 ± 0.0860
Cunefare *et al*.^7^ without retraining	**0.9783 ± 0.0196**	0.5549 ± 0.1916	0.7666 ± 0.2523
Bergeles *et al*.^4^	0.9090 ± 0.0400	0.6770 ± 0.1690	0.7930 ± 0.1689

**Figure 7:**
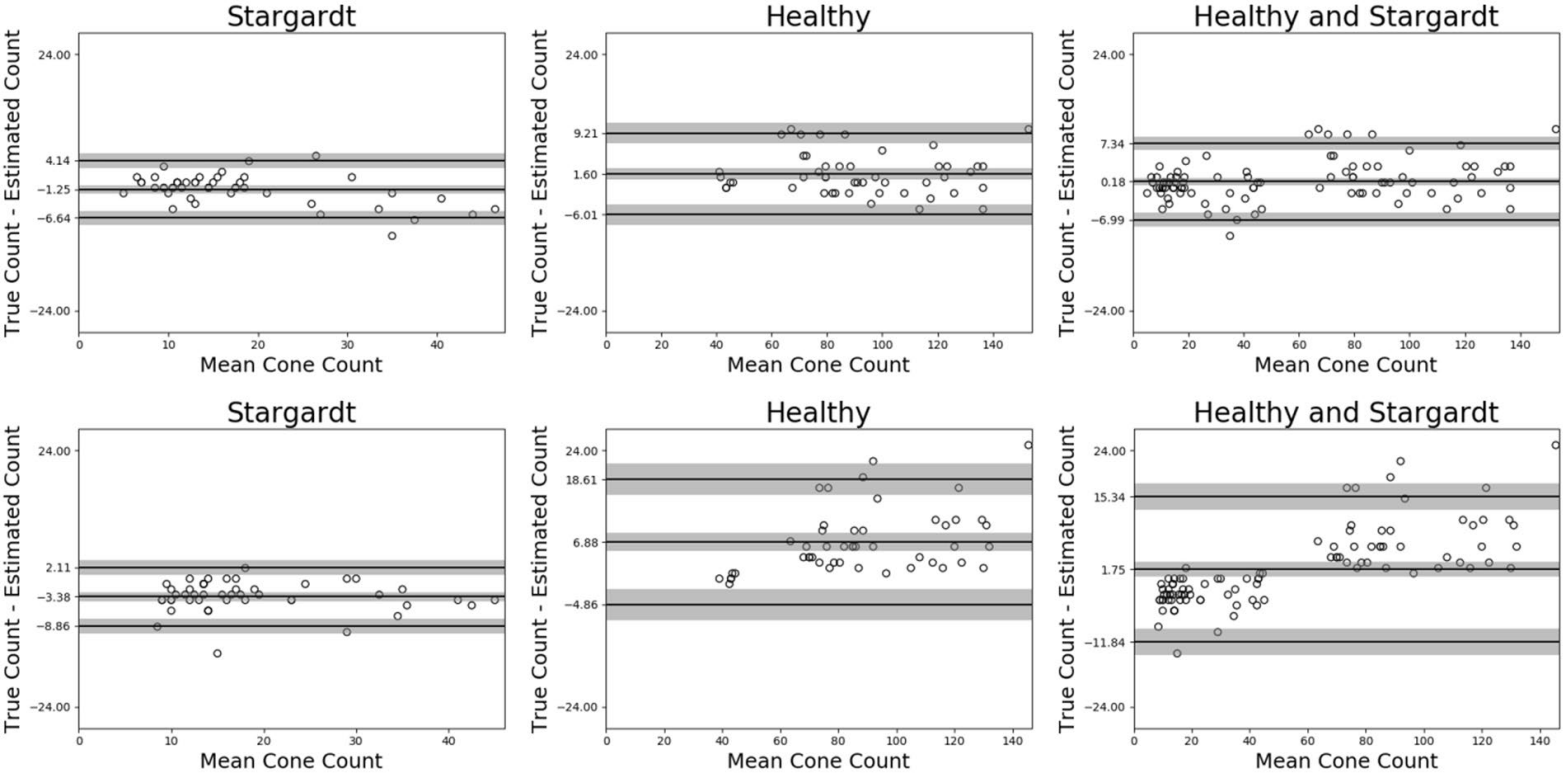
The top row shows Bland-Altman plots for the proposed network, while the bottom row shows the same plots for the retrained convolutional network. In all plots we see that the proposed network’s limits of agreement, and corresponding 95% confidence intervals, are narrower indicating the proposed networks is more accurate when extracting cone number from AOSLO images.

**Figure 8:**
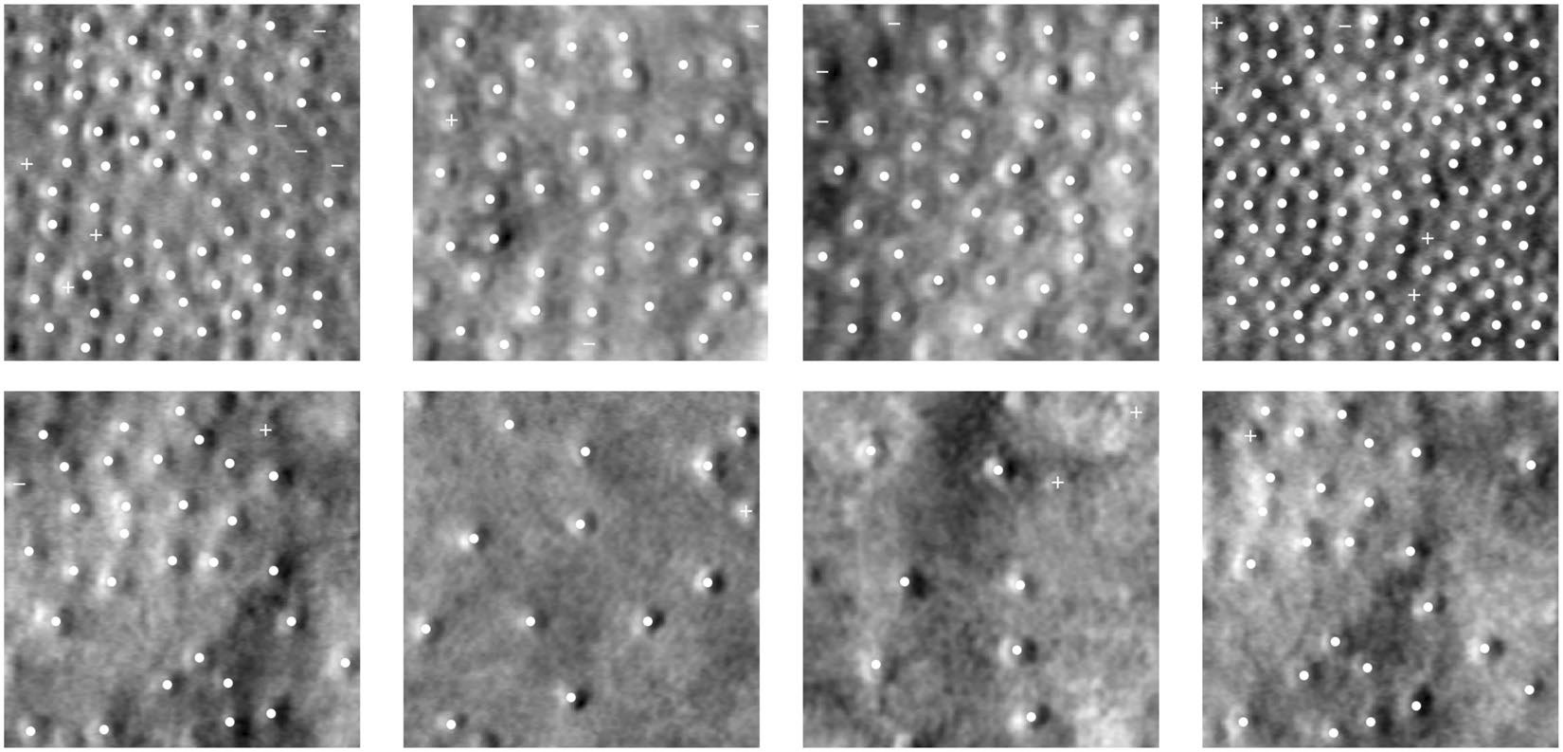
Top row: healthy retinas. Bottom row: retinas afflicted Stargardt disease. True positives are denoted as circles (○), false positives as pluses (+), and false negatives as minuses (−).

The improved Dice score of the proposed method highlights that the network produces more robust estimations of biomarkers, such as cone number. The ability of the proposed approach to deal with varied images allowed our network to estimate cone number in AOSLO split-detection images better than the state-of-the-art convolutional approach. In the proposed approach the 95% confidence interval for the average difference in cone counts was 0.18 ± 1.47, with an upper limit-of-agreement (ULOA) given by 7.34 ± 2.55, and a lower limit-of-agreement (LLOA) of $-6.99\pm 2.55$; for the retrained convolutional approach we found an average difference of $1.75\pm 2.70$, an ULOA of 15.34 ± 4.83, and a LLOA of −11.84 ± 4.83. The proposed approach has tighter limits-of-agreement, and narrower confidence intervals. This is further evidence our approach can be used to automate, with a high degree of accuracy, the tedious manual imaging processing currently required in AOSLO imaging studies (see Figs 7 and 8).

In addition to being more accurate, the method proposed here is also substantially faster. The average time elapsed for single image processing is 0.94 second, versus 7.9 seconds for the deep learning approach of Cunefare *et al*. This 8× speed up significantly reduces the computational burden of processing year-spanning longitudinal studies that capture AOSLO images. Both algorithms were evaluated on a laptop computer with 8 GB RAM, i 7 processor, and a 4 GB NVidia Geforce GTX 1050 GPU.

### Performance on Unseen Pathologies

**Figure 9:**
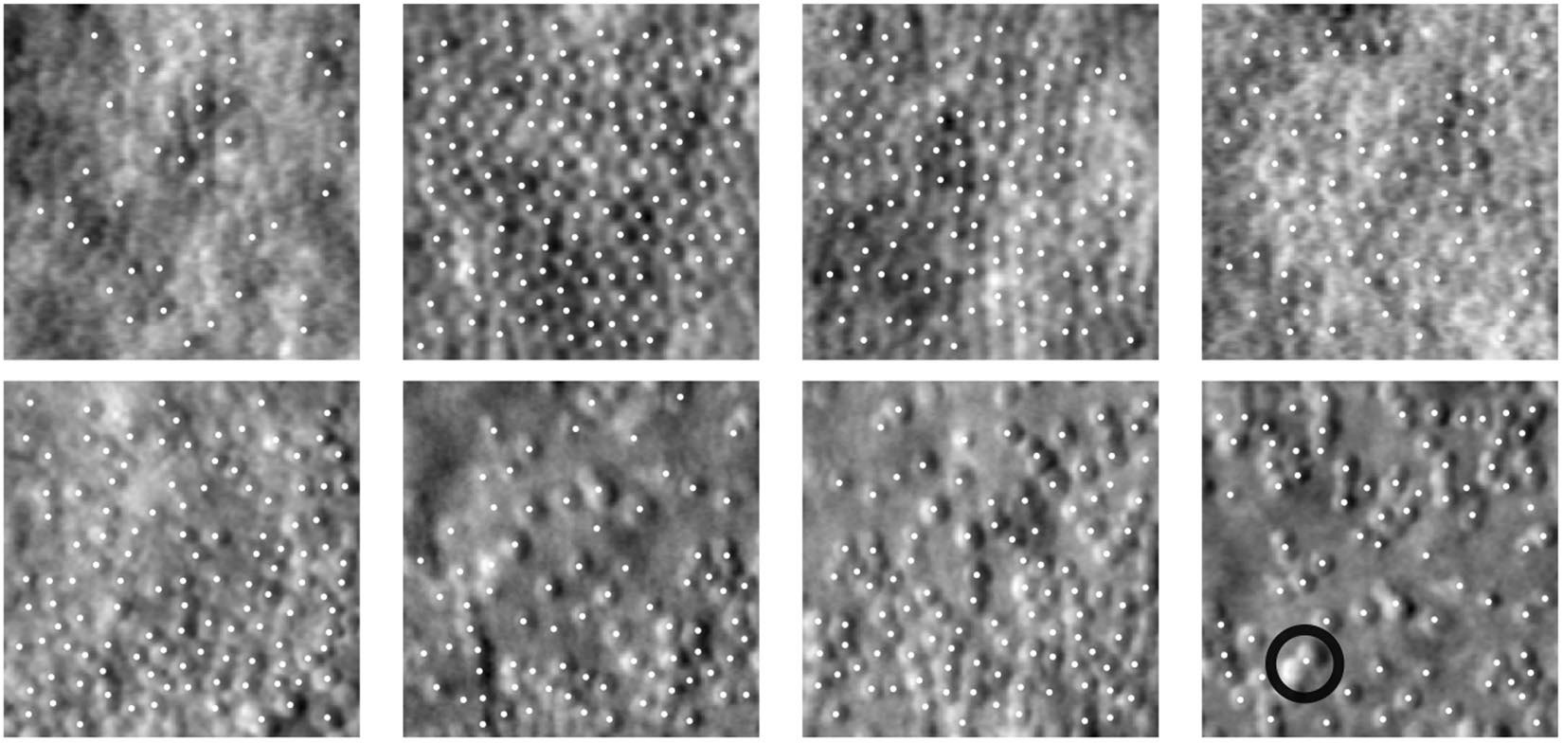
Applying the proposed approach to unseen pathologies. The top row shows the estimated cone locations in images of retinas with RP. The bottom row shows estimated locations for images of retinas with ACHM. The black circle highlights a pair of cones which are marked as a single cone.

We are confident of the network’s ability to generalise to handle unseen retinal conditions, as it was able to successfully locate cones in images of retinas with RP and ACHM, despite never being trained on such conditions. Cone topology and appearance in both of these conditions significantly differs from the healthy and Stargardt images, which the network was trained on. We found, qualitatively, that the network could be successfully applied to both conditions (see Fig. 9). We do not include Dice scores for these images as we lack ground truth locations. The ability to successfully generalise to unseen pathologies is evidence that the developed method is an appropriate framework, capable of locating cones across a range of pathologies.

Our approach did miss cones in some images. In ACHM images, pairs of cones may be marked as a single cone. This can be attributed to cones which appear to merge, a feature which does not occur in Stargardt or healthy images. In images of RP retinas, some cones which an experienced marker could find are missed, though image quality makes cone localisation challenging even for an experienced marker. These performance issues are addressed in the discussion.

## Discussion

Through the use of MDRNN architectures, this paper presents the most robust automatic cone detection algorithm to date. The proposed approach is capable of dealing with highly disparate inputs, as evidenced by its strong performance on images of healthy retinas and those afflicted by Stargardt disease. Furthermore, a strong performance was maintained when validating against images of retinas, with pathologies the network had never been trained on. The proposed approach is significantly faster than the state-of-the-art, further enabling its use in the clinical environment. By utilising MDLSTM blocks, we developed a robust clinical tool with low computational demands, which can reduce the labour intensive and time consuming image analysis associated with AOSLO.

The proposed method can also automatically estimate cone shape, due to the intermediate segmentation it produces. This is an important aspect of the approach as it is well understood from histology that photoreceptors may change shape due to pathology. Estimations of cone shape from AOSLO imaging alone therefore become potentially valuable biomarkers for early diagnosis^34^. Due to a lack of ground truth cone shapes, we mention this as a discussion point and intend to investigate it in future work.

Due to the strong performance across highly disparate images, we are confident that performance issues can be addressed by retraining with more data. The proposed method, as is, can deal with healthy datasets and Stargardt datasets. Within each of these populations their is a lot of variation, which the network is able to handle. Moreover, it could be successfully applied to images with significantly different cone shape, topology, and greatly reduced image quality. On this basis, we aim to collect more data from a wider range of pathologies, and retrain the network described herein. We believe this will suffice to construct a framework which can handle all pathologies currently considered in AOSLO imaging studies.

There are still important challenges which need to be overcome, before the method can be used in a fully automated manner, without human supervision. It is important to conduct a rigorous clinical evaluation of the method, considering a wider range of pathologies and AOSLO imaging devices to ensure robust performance. Although we do not anticipate any major theoretical challenges in translating the method across AOSLO imaging devices based on Scoles *et al*.^2^, as all follow the same design, use the same software, and are made from components from the same manufacturer. We believe the proposed approach would also work for variations of the imaging system, but may need to be retrained. As we collect more data from the clinic, such rigorous comparisons may take place, and we hope to demonstrate further the robustness of the proposed approach. However, the tool is currently packaged in a format which allows it to be used immediately in AOSLO imaging labs, in a semi-automated manner, with human supervision.

In the future we hope to compare the performance of state-of-the-art, convolutional, segmentation frameworks, such as PSPNet^35^, to our MDLSTM approach. A large body of literature in automated medical image analysis relies on convolutional frameworks, whilst we feel recurrent architectures implicitly address many of the problems convolutional approaches face. For example, one of the strengths of PSPNet over other convolutional segmentation frameworks, is its ability to enforce globally consistent segmentations. This ability, however, is encoded into recurrent frameworks *a priori*. By providing access to our software, we encourage others to contrast the performance of recurrent architectures to state-of-the-art convolutional frameworks.
